# Evidence of compensation for mitochondrial reactive oxygen species increase in *Caenorhabditis briggsae* cytoplasmic-nuclear hybrids

**DOI:** 10.17912/micropub.biology.001319

**Published:** 2024-09-23

**Authors:** Emma Hernandez, Joseph Ross, Laurent Dejean

**Affiliations:** 1 Department of Biology, California State University, Fresno, Fresno, California, United States; 2 Department of Chemistry and Biochemistry, California State University, Fresno

## Abstract

Hybrid offspring dysfunction in cytoplasmic-nuclear hybrids (cybrids) implies that one parent's mitochondrial genome is incompatible with the nuclear genome of the other parent. In *Caenorhabditis briggsae* , cybrids exhibit increased mitochondrial reactive oxygen species (ROS). In this study, we measured the specific activity of markers for mitochondrial abundance (citrate synthase) and antioxidant enzyme response (catalase) in four *C. briggsae* cybrid lines. An increase of catalase expression but not in mitochondrial abundance was found in dysfunctional cybrids. This suggests that organisms might compensate for some genetic incompatibilities by modulating gene expression of key oxidative stress enzymes such as catalase.

**Figure 1. Comparison of cybrid and parental strain enzyme activities:**
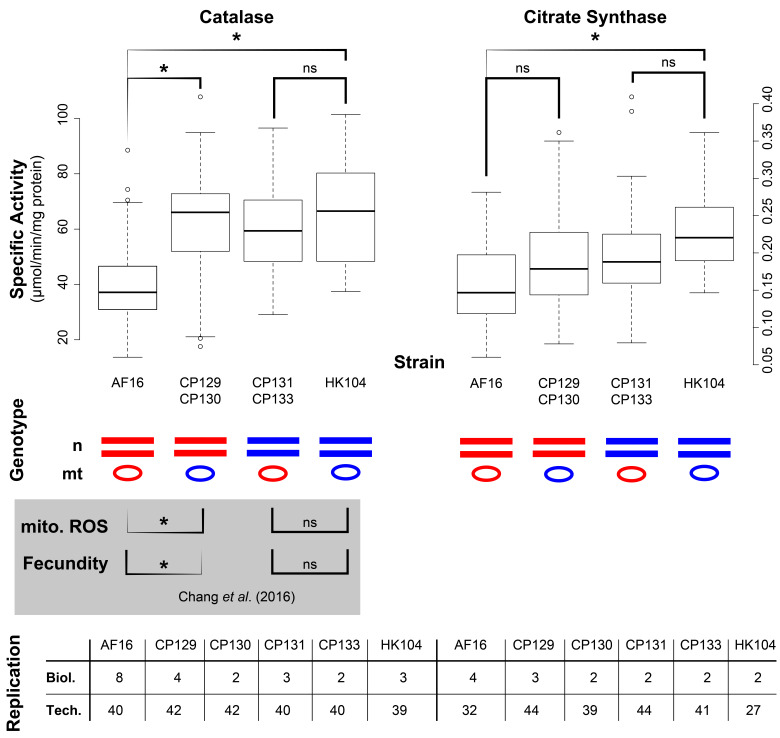
The specific activities of two enzymes (catalase, left panel; citrate synthase, right panel) were measured in biological and technical replicates of four genotypes of strains. Horizontal lines represent nuclear (“n”) chromosomes (genotype) in this diploid species; ovals represent mitochondrial (“mt”) genomes (mitotype). Parental strains are AF16: red n and red mt, and HK104: blue n and blue mt cybrids are CP129 and CP130 (AF16 n and HK104 mt) and CP131 and CP133 (HK104 n and AF16 mt). Boxplots show the mean (thick horizontal line), 25 ^th^ and 75 ^th^ percentile data (bottom and top of the box), 5 ^th^ and 95 ^th^ percentiles (whiskers) and outlier data (circles). Statistical comparisons used one-factor ANOVA: * p < 0.05, ns p >= 0.05. The thicker end of a bracket indicates the larger value in each significant comparison. A summary of statistical comparisons of mitochondrial ROS amount and fecundity of the same strains in a prior publication is provided in the gray box beneath the plot (Chang et al., 2016). For catalase and citrate synthase comparisons, because the biological replicate cybrid values were not statistically significantly different from each other, they were combined for the comparison to the parental strain. The table at the bottom shows the number of biological replicates (total number of independent total protein extracts from each strain) and technical replicates (total number of independent enzyme activity measurements of all the biological replicate extracts).

## Description

Speciation involves reduced gene flow between populations, which ultimately leads to reproductive isolation. Reduction in gene flow and increase in divergence can be attributed to differences in geographic locations, climates, or behaviors, for example (Coyne et al., 2004) . Copulation between two reproductively isolated species results in hybrid dysfunction: reduced fitness in comparison to the parental populations. Many incompatible alleles that cause hybrid inviability or sterility have been discovered. Among these, mitochondrial-nuclear genetic incompatibility has been consistently observed across the plant, animal, and fungal kingdoms (Burton et al., 2006; Gershoni et al., 2009) . Suboptimal activity of mitochondrial oxidative phosphorylation (OXPHOS) has been observed across several species, all of which exhibit a decrease in fecundity *e.g.* (Chang et al., 2016; Edmands and Burton, 1999; Gibson et al., 2013; Meiklejohn et al., 2013) . Such trends suggest that a change in mitochondrial function is often associated with hybrid dysfunction, hybrid incompatibility and speciation (Gershoni et al., 2009) .

Like its close relative *Caenorhabditis elegans* , the microscopic nematode *C. briggsae* possesses several features making it a suitable model system to study the phenomenon of hybrid incompatibility: small size, short generation time, and a large brood size (Gupta et al., 2007) . *C. briggsae* also comprises males and self-fertile hermaphrodites. Mating two genetically distinct populations in the parental (P0) generation produces F1 hybrids. Serial backcrossing of F1 and subsequent generation hybrid hermaphrodites to males of the paternal P0 population produces cytoplasmic-nuclear hybrids (cybrids) that have the mitochondrial genome of the P0 maternal population and the nuclear genome of the P0 paternal population. This genetic composition is often used to identify cytonuclear genetic incompatibilities, where cybrids are less fit than the parental populations. Caenorhabditis nematodes are widely used for exploring such mitonuclear interactions (Estes et al., 2023) . Of relevance here, *C. briggsae* cybrids show a decrease in fecundity and an increase in mitochondrial reactive oxygen species (ROS) staining, likely resulting from mitonuclear incompatibility (Chang et al., 2016) .

Correlative evidence suggests that organisms might use multiple mechanisms to adapt to mitochondrial dysfunction. An increase in mitochondrial genome copy number occurs in mutant *C. elegans* lines with increased ROS (Wernick et al., 2019) and in *C. elegans* cybrids (Song et al., 2020) . No change in mitochondrial citrate synthase specific activity, which is generally used as a mitochondrial mass index or proxy (Dejean et al., 2002; Hutter et al., 2004) , was observed in dysfunctional copepod hybrids with mismatched mitochondrial and nuclear genomes (Ellison and Burton, 2006) . In one study of human cybrid cells, increased ROS was correlated both with an increase in mitochondrial mass and in mitochondrial genome copy number, but catalase activity was not investigated (Wei et al., 2001) . These findings further suggest the possibility that organisms might compensate for mito-nuclear mismatch and increased ROS production through changes in antioxidant gene activity. Thus, it is possible that gene expression regulation could compensate for increased mitochondrial ROS in cybrids.

The long-term goal of this effort is to mechanistically connect cybrid genotypes to these organismal dysfunction phenotypes. ROS are damaging to biomolecules and are generated by mitochondria during OXPHOS. Cells manage ROS levels in several ways, including the activity of enzymes like superoxide dismutases (SOD) and catalases, as reviewed in (Balaban et al., 2005) . ROS production varies among *C. briggsae* wild isolates (Estes et al., 2011) , can be experimentally evolved to increase in *C. elegans* (Joyner-Matos et al., 2013) , and high-ROS *C. elegans* mutants can be experimentally evolved to reduce ROS production (Wernick et al., 2019) . Others have suggested that variation among *C. briggsae* wild isolate ROS levels might be countered by evolutionary pressures to reduce ROS levels in some environments (Hicks et al., 2012) . These prior findings suggest that restoration of mitochondrial function can occur, perhaps through increased ROS scavenging by enzymes. Mitochondrial genotype is also known to influence ROS production in hybrids, as reviewed in (Hill et al., 2019) , and hybridization in sunfish results in increased ROS generation (Du et al., 2017) . Thus, it is feasible that mito-nuclear incompatibilities increase ROS levels and that cybrids can compensate for this deleterious effect.

In the present study, we sought to further explore the biochemical effects of mitochondrial and nuclear genome incompatibilities on the antioxidant catalase and on the mitochondrial abundance enzyme marker citrate synthase (Dejean et al., 2002; Hutter et al., 2004) in our *C. briggsae* cybrids. These measurements were performed on total protein lysates from the two parental strains (AF16 and HK104), two biological replicate cybrid strains with the AF16 genotype and the HK104 mitotype (CP129 and CP130), and two cybrid strains with the HK104 genotype and the AF16 mitotype (CP131 and CP133). Catalase activity was measured using the protocol described in (Rafikov et al., 2014) ; and citrate synthase activity was determined using the protocol described in (Moriyama and Srere, 1971) . These assays were performed in the presence of a large excess of substrates. Catalase and citrate synthase specific activities measured in parental strains and cybrids during this study are reported in Figure 1 .

To identify effects related to a change in mitotype, a parental strain is typically compared to the cybrid with the same nuclear genotype but the mitotype of the cybrid’s other parental strain. Before comparing the two biological replicate cybrids to each P0 parental strain, we used Student’s t-tests to determine whether the replicate cybrid phenotypes were different from each other. In both cases, the cybrids were not significantly different (p >= 0.05), so their data were combined for comparison with the parental strain. CP131 and CP133 constituted our negative controls, as their fecundity and mitochondrial ROS levels were previously shown to be similar to HK104 ( Figure 1, gray box). However, CP129 and CP130 have decreased fecundity and increased mitochondrial ROS levels compared to AF16 ( Figure 1, gray box).

We observed two different trends when comparing the parental strains to their respective mitotype cybrids. Catalase specific activity was significantly increased in cybrids with an AF16 genotype and a HK104 mitotype (CP129 and 130) vs. the AF16 parental strain (p < 0.05, One-factor ANOVA, n=40–42 technical replicates). No difference was observed in the reciprocal cybrid strains with an HK104 nuclear genotype (CP131 and 133) vs. the HK104 parental strain (p >= 0.05, One-factor ANOVA, n=40–42 technical replicates; Figure 1, left panel). No significant difference was observed when comparing the citrate synthase specific activity in cybrids with an AF16 genotype (i.e. CP129 and 130) vs. the AF16 parental strain, or in cybrids with an HK104 genotype (i.e. CP131 and 133) vs. the HK104 parental strain (p > 0.05, One-factor ANOVA, n=27–44 technical replicates; Figure 1, right panel).

In our study, we found that catalase activity is increased in *C. briggsae* cybrids that have higher ROS levels, and we found no significant difference in mitochondrial abundance in the cybrids as determined by citrate synthase activity. This suggests the possibility that one mechanism for organisms to cope with increased ROS is to compensate by increasing antioxidant gene activity. As discussed previously (Chang et al., 2016) , it is not particularly remarkable that one cybrid genotype (AF16 nuclear genome and HK104 mitochondria) shows increased ROS and catalase activity where the reciprocal genotype does not (HK104 nuclear genome and AF16 mitochondria). This observation suggests that AF16 nuclear alleles are sensitive to mitotype, while the HK104 alleles are not as sensitive. Such asymmetric effects are known, including in other *C. briggsae* hybrids (Haddad et al., 2018) .

Our data are in accord with some past findings that mitochondrial abundance does not change in hybrid lines (Ellison and Burton, 2006) , but others have shown that mitochondrial mass increases in human cybrid cell lines (Silva et al., 2013) . Many human cybrid cell lines produce increased ROS, including in models of pulmonary hypertension (Silva et al., 2013) and neurological, metabolic and connective tissue symptoms (Schaefer et al., 2022) . A correlation of increased ROS and increased catalase expression has been demonstrated in human cybrid cell lines related to Parkinson’s disease (Cassarino et al., 1997) and other pathological mitochondrial variants (Park et al., 2009) . Importantly, studies of human mitochondrial genetic disorders have also identified such correlations between ROS and catalase, in mitochondrial encephalomyopathy with lactic acidosis and stroke-like episodes (Vives-Bauza et al., 2006) , although a study of Leber hereditary optic neuropathy human cybrid cell lines identified increased ROS but no change in catalase expression (Floreani et al., 2005) .

Thus, a consensus interpretation of the data suggests that organisms experiencing mitochondrial dysfunction, either by *de novo* mitochondrial mutation or incompatible mitochondrial and nuclear genomes, use multiple not mutually exclusive mechanisms to compensate for increased ROS production. In the *C. briggsae* cybrids, we identified a correlation between increased ROS and increased catalase activity but not a change in mitochondrial abundance. These findings motivate more work in *C. briggsae* to explore the biochemical and cell biological connections between mitochondrial dysfunction and organismal phenotypes like reduced fecundity, as well as to explore the role of mitochondria in the genetic basis of hybrid dysfunction and potentially speciation.

## Reagents

**Table:** 

Strain	Genotype	Available From
AF16	*C. briggsae* wild isolate	CGC*
HK104	*C. briggsae* wild isolate	CGC
CP129	*C. briggsae* AF16 x HK104 cybrid	Chang et al. (2016)
CP130	*C. briggsae* AF16 x HK104 cybrid	Chang et al. (2016)
CP131	*C. briggsae* HK104 x AF16 cybrid	Chang et al. (2016)
CP133	*C. briggsae* HK104 x AF16 cybrid	Chang et al. (2016)

* *Caenorhabditis* Genetics Center

## References

[R1] Balaban RS, Nemoto S, Finkel T (2005). Mitochondria, oxidants, and aging.. Cell.

[R2] Burton RS, Ellison CK, Harrison JS (2006). The sorry state of F2 hybrids: consequences of rapid mitochondrial DNA evolution in allopatric populations.. Am Nat.

[R3] Cassarino DS, Fall CP, Swerdlow RH, Smith TS, Halvorsen EM, Miller SW, Parks JP, Parker WD Jr, Bennett JP Jr (1997). Elevated reactive oxygen species and antioxidant enzyme activities in animal and cellular models of Parkinson's disease.. Biochim Biophys Acta.

[R4] Chang CC, Rodriguez J, Ross J (2015). Mitochondrial-Nuclear Epistasis Impacts Fitness and Mitochondrial Physiology of Interpopulation Caenorhabditis briggsae Hybrids.. G3 (Bethesda).

[R5] Coyne JA, Orr HA. 2004. Speciation. Oxford University Press.

[R6] Dejean L, Beauvoit B, Bunoust O, Guérin B, Rigoulet M (2002). Activation of Ras cascade increases the mitochondrial enzyme content of respiratory competent yeast.. Biochem Biophys Res Commun.

[R7] Du SNN, Khajali F, Dawson NJ, Scott GR (2017). Hybridization increases mitochondrial production of reactive oxygen species in sunfish.. Evolution.

[R8] Edmands S, Burton RS (1999). CYTOCHROME C OXIDASE ACTIVITY IN INTERPOPULATION HYBRIDS OF A MARINE COPEPOD: A TEST FOR NUCLEAR-NUCLEAR OR NUCLEAR-CYTOPLASMIC COADAPTATION.. Evolution.

[R9] Ellison CK, Burton RS (2006). Disruption of mitochondrial function in interpopulation hybrids of Tigriopus californicus.. Evolution.

[R10] Estes S, Coleman-Hulbert AL, Hicks KA, de Haan G, Martha SR, Knapp JB, Smith SW, Stein KC, Denver DR (2011). Natural variation in life history and aging phenotypes is associated with mitochondrial DNA deletion frequency in Caenorhabditis briggsae.. BMC Evol Biol.

[R11] Estes S, Dietz ZP, Katju V, Bergthorsson U (2023). Evolutionary codependency: insights into the mitonuclear interaction landscape from experimental and wild Caenorhabditis nematodes.. Curr Opin Genet Dev.

[R12] Floreani M, Napoli E, Martinuzzi A, Pantano G, De Riva V, Trevisan R, Bisetto E, Valente L, Carelli V, Dabbeni-Sala F (2005). Antioxidant defences in cybrids harboring mtDNA mutations associated with Leber's hereditary optic neuropathy.. FEBS J.

[R13] Gershoni M, Templeton AR, Mishmar D (2009). Mitochondrial bioenergetics as a major motive force of speciation.. Bioessays.

[R14] Gibson JD, Niehuis O, Peirson BR, Cash EI, Gadau J (2013). Genetic and developmental basis of F2 hybrid breakdown in Nasonia parasitoid wasps.. Evolution.

[R15] Gupta BP, Johnsen R, Chen N (2007). Genomics and biology of the nematode Caenorhabditis briggsae.. WormBook.

[R16] Haddad R, Meter B, Ross JA (2018). The Genetic Architecture of Intra-Species Hybrid Mito-Nuclear Epistasis.. Front Genet.

[R17] Hicks KA, Howe DK, Leung A, Denver DR, Estes S (2012). In vivo quantification reveals extensive natural variation in mitochondrial form and function in Caenorhabditis briggsae.. PLoS One.

[R18] Hill GE, Havird JC, Sloan DB, Burton RS, Greening C, Dowling DK (2018). Assessing the fitness consequences of mitonuclear interactions in natural populations.. Biol Rev Camb Philos Soc.

[R19] Hutter E, Renner K, Pfister G, Stöckl P, Jansen-Dürr P, Gnaiger E (2004). Senescence-associated changes in respiration and oxidative phosphorylation in primary human fibroblasts.. Biochem J.

[R20] Joyner-Matos J, Hicks KA, Cousins D, Keller M, Denver DR, Baer CF, Estes S (2013). Evolution of a higher intracellular oxidizing environment in Caenorhabditis elegans under relaxed selection.. PLoS One.

[R21] Meiklejohn CD, Holmbeck MA, Siddiq MA, Abt DN, Rand DM, Montooth KL (2013). An Incompatibility between a mitochondrial tRNA and its nuclear-encoded tRNA synthetase compromises development and fitness in Drosophila.. PLoS Genet.

[R22] Moriyama T, Srere PA (1971). Purification of rat heart and rat liver citrate synthases. Physical, kinetic, and immunological studies.. J Biol Chem.

[R23] Park JS, Sharma LK, Li H, Xiang R, Holstein D, Wu J, Lechleiter J, Naylor SL, Deng JJ, Lu J, Bai Y (2009). A heteroplasmic, not homoplasmic, mitochondrial DNA mutation promotes tumorigenesis via alteration in reactive oxygen species generation and apoptosis.. Hum Mol Genet.

[R24] Rafikov R, Kumar S, Aggarwal S, Hou Y, Kangath A, Pardo D, Fineman JR, Black SM (2013). Endothelin-1 stimulates catalase activity through the PKCδ-mediated phosphorylation of serine 167.. Free Radic Biol Med.

[R25] Schaefer PM, Scherer Alves L, Lvova M, Huang J, Rathi K, Janssen K, Butic A, Yardeni T, Morrow R, Lott M, Murdock D, Song A, Keller K, Garcia BA, Francomano CA, Wallace DC (2022). Combination of common mtDNA variants results in mitochondrial dysfunction and a connective tissue dysregulation.. Proc Natl Acad Sci U S A.

[R26] Silva DF, Selfridge JE, Lu J, E L, Roy N, Hutfles L, Burns JM, Michaelis EK, Yan S, Cardoso SM, Swerdlow RH (2013). Bioenergetic flux, mitochondrial mass and mitochondrial morphology dynamics in AD and MCI cybrid cell lines.. Hum Mol Genet.

[R27] Song Y, Wang Y, Li Y, Wang L, Zhang W, Cheng J, et al., Zhu Z. 2020. The whole transcriptome regulation as a function of mitochondrial polymorphisms and aging in Caenorhabditis elegans. Aging (Albany NY) 12(3): 2453-2470. 10.18632/aging.102754 PMC7041728 32019902

[R28] Vives-Bauza C, Gonzalo R, Manfredi G, Garcia-Arumi E, Andreu AL. 2006. Enhanced ROS production and antioxidant defenses in cybrids harbouring mutations in mtDNA. Neurosci Lett 391(3): 136-41. 10.1016/j.neulet.2005.08.049 16165271

[R29] Wei YH, Lee CF, Lee HC, Ma YS, Wang CW, Lu CY, Pang CY (2001). Increases of mitochondrial mass and mitochondrial genome in association with enhanced oxidative stress in human cells harboring 4,977 BP-deleted mitochondrial DNA.. Ann N Y Acad Sci.

[R30] Wernick RI, Christy SF, Howe DK, Sullins JA, Ramirez JF, Sare M, Penley MJ, Morran LT, Denver DR, Estes S (2019). Sex and Mitonuclear Adaptation in Experimental Caenorhabditis elegans Populations.. Genetics.

